# Mrs. Fawcett and Socialism

**Published:** 1890-11-15

**Authors:** 


### The Doctor's Armchair.

### MRS. FAWCETT AND SOCIALISM.

Few women can better stand comparison with men than Mrs. Fawcett. She is delivering a course of lectures on social and economic subjects at the North London High School for Girls, and it was the writer's privilege last week to hear one of them. Mrs. Fawcett has a firm, sober, and kindly face, which pleases and inspires confidence. She has no resemblance whatever to the caricatures of the masculine woman which used to be popular a dozen years ago. On the contrary, her appearance is that of a capable, helpful, intelligent, and motherly housewife of forty-five. She is probably older than that, but she does not look it. When lecturing, Mrs. Fawcett speaks in clear, musical tones, and with measured distinctness. Her face is not vivacious, but it is expressive; her manner corresponds with her face. She gives the impression, almost immediately, that her temper is fair, but that her convictions are so strong as to need much reining in, in order that they may run level with her sense of justice. One has a feeling, in listening to her expositions, that her fellow-women ought to be proud of her, and that the men of England should also feel a sense of pride in the fact that she is an English woman, and that she represents, in an exalted degree, the characteristics and peculiarities which distinguish the women of Britain from those of other countries. If this judgment be correct we men of England have good reason to be satisfied with our sisters, our daughters, and our wives.

Mrs. Fawcett lectured on Socialism. The subject is one to which some of us have given very little attention; we have considered the theories of the more advanced socialists to be, if a plain word may be used, stupid; and men who work hard, even though they may try to think hard too, cannot afford to waste their thinking leisure on subjects that are neither practical nor mentally stimulating. Mrs. Fawcett seems to think, however, that the socialist writers have established a claim to attention; and having made up her mind to deal with them, she does it in a thoroughly business-like and conclusive fashion. What do the socialists want? she asks herself and her hearers. They want to make every man exactly the equal of every other man: individualism is to cease and determine: collectivism is to be the daily work, the religion, the politics, and the be-all and end-all of everything and everybody. To our thinking a sufficient answer to this theory is that it is a fundamental contradiction of human nature. It and its propounders are worth so much attention, and no more, than an anatomist would be who should affirm that the proper organ for walking with is the head; and that a man's feet, instead of being on the ground, ought to be in the air. Mrs. Fawcett is more civil to the socialists than we are inclined to be; but then she has more leisure, and leisure is greatly conducive to suavity of manners.

Has the world had any experience of socialism? asked the lecturer. It has had a good deal of experience of one kind or another; and that experience has been sufficient to enable the thoughtful student to arrive at this conclusion—that whilst individualism means freedom, socialism means slavery. The case of Sparta, among others, was cited in illustration; and those who are inclined to be enamoured of the paper plausibilities of the socialist writers will do well to spend a few weeks in reading and considering the history of the Spartan State. Mrs. Fawcett, with a fine practicality, pointed out very clearly that whilst state socialism is illogical, impossible, and absurd, there may be occasions when it is not only right but the highest wisdom for individuals of different ranks and possessions to band themselves together in voluntary associations of brotherhood and equality for the purposes of practical work. The early Christians did this for a time, and found it to work well in their peculiar circumstances. The Moravians have done and still do the same. Three or four partners in a trading enterprise associate themselves together for the purposes of the business; but their object in doing so is not the good of each other or the honour and glory of the firm. Individualism is the ultimate ruling motive in each partner's mind; and he only joins the firm because he hopes thereby to serve his own interests better than he would by carrying on business alone. It is plain, in fact, that self-interest is the ruling motive of man as man everywhere; and it is as clear as noonday that the Creator of man intended it to be so. There are the best of all reasons why man should have been made in this way; and the chief of them is this, that being so made, every man is forced by an inward compulsion to take care of himself, and does not wait for other people to take care of him.

Some good folks ask what Christianity has to say to such a view as this? Christianity is something superadded to human nature. But inasmuch as it is a divine religion, it is entirely reasonable, and recognises the actual condition of the natural man. We say that the first and deepest of all human instincts is for a man to take care of himself, and the next is that he shall take care of those who are nearest to him in blood and other human relationships. What does St. Paul, as a representative of Christianity, say on this point? He says, "He that neglecteth to provide for his own, and especially for those of his own household, hath denied the faith, and is worse than an infidel." But does not Christianity teach that all Christians are brothers and equals? It does; and the meaning of this is that each individual Christian man is to feel as a brother, and to acknowledge the brotherhood not only of every other Christian man, but of every other man of every sort. Would that Christian bachelor who had a thousand a-year, and no children, prove his brotherhood by going to his neighbour who had twelve hundred a-year, and twelve children, and demanding a hundred a-year from him on the ground that their two incomes should be equal? Or would even the man with the large family be acting in the spirit of a brother if he compelled his bachelor neighbour by force to give up half his income to him on the ground of family necessities? Christianity teaches brotherhood, but it is voluntary brotherhood; and it teaches that each man shall of his own will act the part of a brother to every other man, which is a very different thing from teaching each man to go about the world with a revolver in his hand, compelling every other man to act the part of a brother towards him. Indeed, if other men took him at his word, and did act the part of brothers, the first thing they would do would be to deprive him of his revolver, and the next to send him to a lunatic asylum.

Mrs. Fawcett mentioned General Booth's new scheme as a departure in the socialistic direction, and as an illustration of the contention that compulsory socialism is, in the last analysis, slavery. General Booth proposes to supply every necessitous man, who asks him, with food. But this is to be on the condition that the man shall work, that his work shall be of a certain definite value, and that General Booth, or his representative, shall be the judge of the value of the work, and of what is to be paid for it. On these conditions every man in the proposed new association shall be entitled to his share of the common stock of necessaries belonging to the association. But this is slavery. It is true it is slavery from which any man can free himself at any moment by his own will, and without risk. Nevertheless, so long as the contract continues, it is a contract of slavery. General Booth is to be the master who shall exercise judgment, free will, and authority. Those who submit themselves to these conditions are for the time being slaves, though, of course, voluntary slaves. General Booth himself sees this clearly, and expresses it frankly. Every man who comes to us, he says in effect, shall eat if he be hungry, first of all; and if he will work he shall continue to eat his full share. But if he will not work he shall not eat; and the decision as to his work, and the value of it, shall rest with the head of the organization, and not with the workers. This, says Mrs. Fawcett, is collectivism as opposed to individualism, and slavery as opposed to freedom. For the purposes of General Booth, and for similar local and temporary objects, it may frequently be the wisest and best course that men can take. But for State and universal purposes compulsory socialism will not only prove to be the death of freedom, but of art, literature, religion, and everything else which constitutes the true and essential greatness of man.

